# Menopausal hormone therapy and the risk of systemic lupus erythematosus and systemic sclerosis: a population-based nested case-control study

**DOI:** 10.1093/rheumatology/keaf004

**Published:** 2025-01-07

**Authors:** Karina Patasova, Marina Dehara, Ängla Mantel, Marie Bixo, Elizabeth V Arkema, Marie Holmqvist

**Affiliations:** Clinical Epidemiology Division, Department of Medicine, Solna, Karolinska Institutet, Karolinska University Hospital T2, Stockholm, Sweden; Clinical Epidemiology Division, Department of Medicine, Solna, Karolinska Institutet, Karolinska University Hospital T2, Stockholm, Sweden; Clinical Epidemiology Division, Department of Medicine, Solna, Karolinska Institutet, Karolinska University Hospital T2, Stockholm, Sweden; Department of Women’s Health, Division of Obstetrics, Karolinska University Hospital, Stockholm, Sweden; Department of Clinical Sciences, Obstetrics and Gynecology, Umeå University, Umeå, Sweden; Clinical Epidemiology Division, Department of Medicine, Solna, Karolinska Institutet, Karolinska University Hospital T2, Stockholm, Sweden; Clinical Epidemiology Division, Department of Medicine, Solna, Karolinska Institutet, Karolinska University Hospital T2, Stockholm, Sweden

**Keywords:** lupus, SSc, menopausal hormone therapy

## Abstract

**Objectives:**

SLE and SSc are more common in women, partly due to differences in female sex hormones. Menopausal hormone therapy (MHT) is widely used to alleviate climacteric symptoms. Here, the relationship between MHT and SLE/SSc was investigated in a nested case-control study.

**Methods:**

Women with SLE or SSc and controls, matched 1 up to 10 on sex, birth year and region, from the general population of Sweden. Data on exposures and potential confounders were obtained from the National Patient and Prescribed Drug Register as well as the Longitudinal Integration Database for Health Insurance and Labour Market Studies. Exposure was defined as the dispensation of any MHT medication prior to the diagnosis/matching. The association between MHT and SLE/SSc, and whether the strength of the association, expressed as odds ratios (OR) and 95% CI, varied by type, route of administration, and duration of use, was assessed using conditional logistic regression, adjusted for education, income and sick leave.

**Results:**

In total, 943 women with SLE and 733 women with SSc were identified between 2009 and 2019. We detected a significant association between MHT use and risk of SLE (OR = 1.3; 95% CI: 1.1–1.6), and SSc (OR = 1.4; 95% CI: 1.2–1.7). Women who had both systemic and local MHT medications dispensed exhibited the highest risk of SLE (OR = 1.9; 95% CI: 1.4–2.7) and SSc (OR = 1.8; 95% CI: 1.2–2.5).

**Conclusion:**

These findings indicate an association between MHT and SLE/SSc, independent of socioeconomic factors, warranting further investigation into the role of exogenous female sex hormones in SLE/SSc pathogenesis.

Rheumatology key messages

- MHT use was associated with 30–90% greater risk of SLE
- MHT use was associated with 40–80% greater risk of SSc.

## Introduction

There are considerable sex imbalances in the immune-mediated diseases SLE and SSc; women comprise >80% of the patient populations [1]. The role of female sex hormones in immune-mediated disease is indirectly supported by disease flares during periods characterized by elevated oestrogen levels, for example during pre-menstrual periods and pregnancy [2].

With diminishing levels of female steroid hormones, the sex gap in SLE prevalence starts to decrease after menopause, shifting the female-to-male ratio from 8:1 to 2:1 [3]. In addition to the immunomodulatory role of sex hormones [1], there is also evidence suggesting a link between exogenous oestrogens, such as menopausal hormone therapy (MHT), and increased risk of SLE as well as mild to moderate flares in established disease [4].

Similar to SLE, strong clinical and immunological differences between men and women have been described in SSc [5, 6]. Given these differences, an influence of sex hormones has been suggested, but only a few studies have directly assessed the impact of sex hormones on SSc pathogenesis [7]. A recently published World Health Organization pharmacovigilance report identified MHT as a potential factor in the development of SSc [8]. Therefore, the aim of this study was to assess the association between MHT and risk of SLE as well as SSc in a large case-control study of women in Sweden.

## Methods

### Study setting

In Sweden, healthcare services are primarily tax-funded and equally accessible to all residents. Patients with SLE and SSc are typically managed and treated at hospital-based specialized rheumatology clinics.

### Data sources

National Patient Register (NPR) collects nationwide longitudinal data on hospitalizations and outpatient visits in non-primary care starting from 1987 and 2001, respectively [9]. Starting in 1997, the International Classification of Diseases (ICD) version 10 was used [9].

The Prescribed Drug Register (PDR) holds information on all dispensed medications from Swedish pharmacies, covering drugs received on an outpatient basis since 2005 [10]. All drugs in the register have an Anatomical Therapeutic Chemical (ATC) code and are assigned dosage (Defined Daily Dose (DDD)) per route of administration [10].

The Total Population Register (TPR) collects longitudinal demographic information on all residents of Sweden, including sex, birth date, civil status, dates of migration and death [9].

Longitudinal Integration Database for Health Insurance and labour Market Studies (LISA) collects data on educational attainment, sick leave and income [11].

### Study design and study population

To study the relationship between the use of MHT and SSc/SLE, we conducted two register-based case-control studies nested in the adult population of Sweden. In this nested case-control study design, diseased cases arising within a predefined cohort (here: the Swedish population) are identified, and for each of them, up to 10 matched controls are randomly selected from the pool of cohort members that did not develop disease at the time of case diagnosis [12]. In each case-control study, women aged 40+ years old with first-ever visits indicating SLE or SSc as main diagnosis between 1 January 2009 and 31 December 2019 were identified from the NPR. The first-ever visit indicating SLE/SSc was used as the index date in both studies. Women with contributory diagnoses of SLE/SSc before the first-ever main diagnosis were excluded from each respective case population. To decrease the risk of misclassification of outcome (SLE/SSc), we required one additional visit indicating SLE/SSc as the main diagnosis within 1–12 months of the first. In the SLE study, we required at least one of the visits to be in rheumatology, dermatology, nephrology, or internal medicine units. In the SSc study, one of the visits had to be at a rheumatology or internal medicine unit. We used ICD10 codes M32 and all its subgroups, excluding drug-induced SLE M32.0, to identify SLE. To identify SSc, we used ICD10 codes M34 and all its subgroups, excluding chemically induced SSc M34.2 (see Supplementary Table S1, available at *Rheumatology* online for ICD codes used).

We identified controls from the TPR and individually matched them by sex, birth year and place of residence in a one-to-ten ratio to their cases. Controls also had to be alive, live in Sweden and without any SLE/SSc diagnoses in the NPR at the second visit date of the cases. Each control was assigned the same index date as their corresponding case.

We excluded all individuals from the study population who had any of the following contraindications for MHT before index date: thromboembolism or prescription of anticoagulants (I80, I81, I82; B01AA, B01AB, B01AE, B01AF, B01AX (anticoagulants)), stroke (I61; I63; I64), ischaemic heart disease (I20–I25), endometrial (C54.1) and breast cancer (C50) (Supplementary Table S1, available at *Rheumatology* online). Moreover, women who started MHT before the age of 40 years old were also excluded from the study, in an attempt to exclude women who were treated with MHT due to other reasons than menopause.

### Definition of exposure

We used ATC codes from the PDR to identify exposure to MHT prior to the index date; G03C (oestrogen); G03D (progestogen, only included if prescribed in combination with oestrogen); G03F (combined oestrogen-progestogen); tibolone (G03CX, used as an alternative for continuous combined oestrogen-progestogen hormone therapy which also has androgenic properties) (Table 1). We classified MHT treatments as systemic or local based on DDDs. Information on DDDs was extracted from PDR, where each ATC code was assigned a unique DDD per route of administration (Table 1). Systemic MHT medications were comprised of oral or transdermal products (i.e. oral tablets, dermal patches and dermal gel), while local drugs included vaginal products (i.e. vaginal creams, rings and pessaries). Information on the calculation of duration of MHT use is provided in Supplementary Methods, available at *Rheumatology* online.

**Table 1.** Anatomical therapeutic chemical codes that were used to identify menopausal hormone therapy use.

MHT treatment	Formulations/derivatives	ATC code
Estrogen only	Estradiol (E2)	G03CA03
	Estriol (E3)	G03CA04
	Conjugated oestrogens	G03CA57
Tibolone only	Tibolone	G03CX01
Progestogen only	Medroxyprogestogen	G03DA02
	Dydrogesterone	G03DB01
	Norethisterone	G03DC02
Estrogen-progestogen combination	Norethisterone combined estrogen	G03FA01
	Medroxyprogestogen combined estrogen	G03FA12
	Dydrogesterone combined estrogen	G03FA14
	Drospirenone combined estrogen	G03FA17

Ever users were defined as women who were dispensed MHT at any time point ≥ 1 day before the index date (Supplementary Table S2, available at *Rheumatology* online). In addition to ever exposure to MHT, we also evaluated associations with MHT type, route of administration and duration of use. Types of MHT medication and route of administration were jointly analysed as a composite variable. Given the history of local oestrogen use among women with systemic oestrogen and oestrogen-progestogen combination treatment, we also considered the route of administration alone (Supplementary Table S2, available at *Rheumatology* online).

### Other variables

We retrieved demographic information from the TPR including date of birth and county of residence at the index date. From LISA, we obtained data on education level (<9, 9–12, 12+ years, unknown), gross income in Swedish Krona (SEK) (adjusted to the 2019 inflation rate) within the calendar year prior to index date and sick leave in the year 2005, coded as a binary variable (yes *vs* no). We used data from 2005 for sick leave to ensure that they were collected before exposure and outcome; sick leave captured later could be a mediator.

### Statistical analyses

The baseline characteristics of the study participants were described as proportions, mean [standard deviation (s.d.)] and median [interquartile range (IQR)] for categorical and continuous variables, accordingly.

The relationships between MHT ever being dispensed and the risk of SLE/SSc were assessed using conditional logistic regression models and expressed as odds ratios (OR) with 95% CI. ORs were used to estimate risk ratios. Models were adjusted for education, gross yearly income and sick leave and were conditional on the matched set to account for matching factors. The main study analyses compared the risk of developing SLE/SSc between MHT ever and never-users.

We stratified by time from the first MHT dispensation to the index date (0–7 years) to investigate whether the relationship between MHT and risk of SLE and SSc varied by time since the first MHT dispensation. To examine whether the risk of SLE and SSc changed depending on age when MHT treatment was initiated, we conducted analyses stratified by age at first MHT dispensation. Additionally, analyses excluding women who first received MHT at the age 40–45 years old were also performed.

We performed three sensitivity analyses to evaluate potential misclassification of MHT: (1) a more stringent definition for MHT was applied, restricting exposure to a minimum of two MHT dispensations in the PDR; (2) given that tibolone has androgenic properties in conjunction with oestrogenic and progestogenic properties, we excluded women who received tibolone before index date; (3) apart from menopausal symptoms, MHT is sometimes prescribed to treat a variety of other conditions, so we excluded non-menopausal indications for MHT to evaluate whether these indications influenced the results [premature ovarian failure (ICD10: E28.3; E89.4), endometriosis (ICD10: N80.0—N80.9), premenstrual dysphoric disorder (ICD10: F32.81) and menstrual migraine (ICD10: G43.82; G43.83); Supplementary Table S1, available at *Rheumatology* online].

Data management and statistical analyses were performed, using packages *gtsummary* [13], *survival* [14] and *sjPlot* [15] in R version 4.2.1 [16].

### Ethics

Our study complies with the Declaration of Helsinki. The study was approved by the Swedish Ethical Review Authority, number 2017/2000-31 and 2020-04529. Informed consent was waived due to the register-based design of the study.

## Results

### SLE and MHT use

We included 943 women with SLE and 8381 matched controls (Table 2, Supplementary Fig. S1, available at *Rheumatology* online). The two groups were comparable with respect to educational level, but women with SLE had markedly lower income (Table 2). The proportion of women on extended sick leave (≥14 days) was higher among women with SLE compared with the control women (Table 2). We observed small differences in the ever *vs* never-use of MHT medications between women with SLE and matched controls (26% *vs* 22%) (Table 3). Results from adjusted conditional logistic regression models suggested a statistically significant association between MHT (ever *vs* never) and SLE (OR = 1.3; 95% CI: 1.1–1.6) (Table 3). Depending on the type of MHT medication and route of administration, point estimates varied between 1.0 and 1.9 (Table 3). A combination of systemic oestrogen and progestogen was associated with 50% increased odds of SLE (OR = 1.5; 95% CI: 1.2–2.0), followed by local oestrogen (OR = 1.3; 95% CI: 1.0–1.6) and lastly by systemic oestrogen (OR = 1.0; 95% CI: 0.6–1.6) (Table 3). The group that dispensed local MHT drugs only had 20% elevated odds of the disease (OR = 1.2; 95% CI: 1.0–1.5), whereas systemic MHT was not associated with SLE (OR = 1.1; 95% CI: 0.8–1.5) (Table 3). Women who were dispensed both local and systemic MHT medications exhibited the highest odds of SLE with an OR of 1.9 (95% CI: 1.4–2.7) (Table 3). Analyses concerning duration of exposure showed that there were no major risk differences between longer *vs* shorter exposure to MHT (<12 *vs* ≥12 months) (Table 3).

**Table 2.** Study participants characteristics at index date

	SLE		SSc	
	Cases, *N* = 943	Controls, *N* = 8381	Cases, *N* = 733	Controls, *N* = 6571
Age (years), Median (IQR)^a^	57 (47–67)	57 (48–67)	63 (54–72)	63 (54–72)
Years of education, *n* (%)				
<9 years	103 (11)	831 (10)	95 (13)	863 (13)
9–12 years	508 (55)	4473 (54)	372 (52)	3399 (53)
12+ years	312 (34)	2943 (36)	242 (34)	2179 (34)
Unknown	20	134	24	130
Gross yearly income (SEK), Median (IQR)^b^	203 346 (137 726–290 204)	232 112 (147 519–322 908)	197 750 (135 039–285 475)	209 350 (136 638–309 107)
Sick leave, *n* (%)				
No	753 (80)	7122 (85)	618 (84)	5650 (86)
Yes	190 (20)	1259 (15)	115 (16)	921 (14)

Years of education and gross yearly income in the calendar year before index date. Sick leave was defined as a history of taking >14 days off work during 2005.

a Interquartile range.

b Swedish krona; 2 missing in controls without systemic lupus erythematosus and systemic sclerosis, respectively.

**Table 3.** Association between menopausal hormone therapy and risk of systemic lupus erythematosus and systemic sclerosis

	Menopausal hormone therapy, ever *vs* never at any time point prior to index date						Menopausal hormone therapy, ever *vs* never, ≥3 years prior to index date					
	SLE			SSc			SLE			SSc		
	Cases, *N* = 943	Controls, *N* = 8381	**OR (95% CI)** ^a^	Cases, *N* = 733	Controls, *N* = 6571	**OR (95% CI)** ^a^	Cases, *N* = 943	Controls, *N* = 8381	**OR (95% CI)** ^a^	Cases, *N* = 733	Controls, *N* = 6571	**OR (95% CI)** ^a^
**Menopausal hormone therapy, *n* (%)**												
Never	699 (74)	6517 (78)	Ref.	485 (66)	4672 (71)	Ref.	750 (80)	6852 (82)	Ref.	518 (71)	4994 (76)	Ref.
Ever	244 (26)	1864 (22)	1.3 (1.1–1.6)	248 (34)	1899 (29)	1.4 (1.2–1.7)	193 (20)	1529 (18)	1.2 (1.0–1.5)	215 (29)	1577 (24)	1.5 (1.2–1.8)
**Type of menopausal hormone therapy, *n* (%)** ^b^												
Systemic estrogen	19 (2.6)	173 (2.6)	1.0 (0.6–1.7)	15 (3.0)	161 (3.3)	1.0 (0.6–1.7)	17 (2.2)	185 (2.6)	0.8 (0.5–1.4)	18 (3.4)	154 (3.0)	1.4 (0.8–2.3)
Local estrogen	139 (17)	1101 (14)	1.3 (1.0–1.6)	143 (23)	1152 (20)	1.3 (1.0–1.6)	99 (12)	862 (11)	1.1 (0.8–1.4)	119 (19)	930 (16)	1.4 (1.1–1.7)
Systemic estrogen + progestogen	72 (9.3)	487 (7.0)	1.5 (1.2–2.0)	76 (14)	488 (9.5)	1.7 (1.3–2.2)	66 (8.1)	414 (5.7)	1.7 (1.3–2.3)	69 (12)	425 (7.8)	1.8 (1.3–2.4)
**Route of administration of menopausal hormone therapy, *n* (%)** ^b^												
Systemic	50 (6.7)	466 (6.7)	1.1 (0.8–1.5)	57 (11)	443 (8.7)	1.4 (1.0–1.8)	56 (6.9)	468 (6.4)	1.2 (0.9–1.6)	65 (11)	426 (7.9)	1.7 (1.3–2.3)
Local	139 (17)	1101 (14)	1.3 (1.0–1.6)	143 (23)	1152 (20)	1.3 (1.0–1.6)	99 (12)	862 (11)	1.1 (0.8–1.4)	119 (19)	930 (16)	1.4 (1.1–1.7)
Systemic + local	55 (7.3)	297 (4.4)	1.9 (1.4–2.7)	48 (9.0)	304 (6.1)	1.8 (1.2–2.5)	38 (4.8)	199 (2.8)	2.1 (1.4–3.1)	31 (5.6)	221 (4.2)	1.5 (1.0–2.3)
**Duration of MHT use, *n* (%)**												
<12 months	74 (7.8)	555 (6.6)	1.3 (1.0–1.7)	76 (10)	590 (9.0)	1.3 (1.0–1.7)	33 (3.5)	334 (4.0)	1.0 (0.7–1.4)	53 (7.2)	380 (5.8)	1.5 (1.1–2.1)
≥12 months	170 (18)	1309 (16)	1.3 (1.1–1.6)	172 (23)	1309 (20)	1.4 (1.2–1.7)	160 (17)	1195 (14)	1.3 (1.1–1.6)	162 (22)	1197 (18)	1.5 (1.2–1.8)

a Odds ratio (OR) and 95% CI were estimated from conditional logistic regression models adjusted for years of education and gross yearly income.

b Systemic administration was defined as oral and transdermal products and local as vaginal products. In the analyses comparing menopausal hormone therapy ever users to never users, all menopausal hormone therapy medications were considered if dispensed before the index date. In the analyses examining ≥3 years prior to the index date, exposure was defined as dispensing menopausal hormone therapy at least 3 years before the index date.

**Figure 1.**
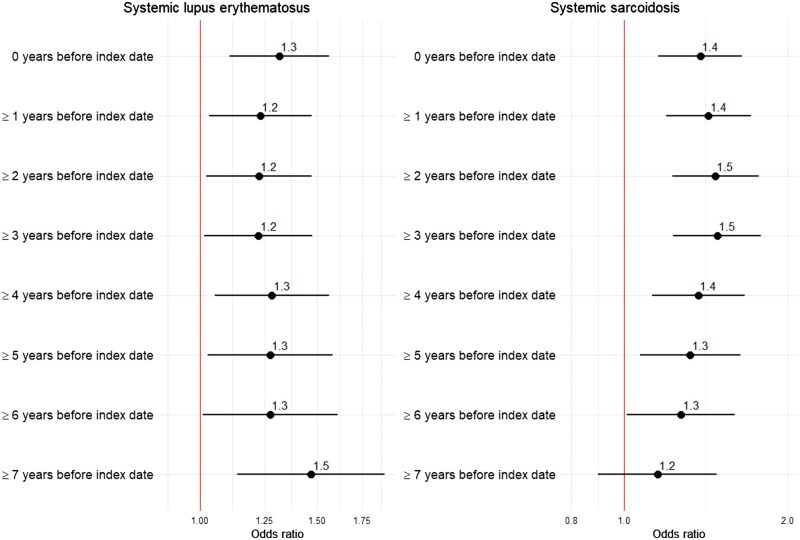
Associations with menopausal hormone therapy according to time between the first dispensation and the index date. Forest plot of odds ratios and their respective 95% CI distributions according to time since first menopausal hormone therapy dispensation. The vertical dashed line denotes odds ratio of 1.0. Conditional logistic models were adjusted for years of education and gross yearly income during the calendar year before index date, sick leave (yes *vs* no) during 2005

**Figure 2.**
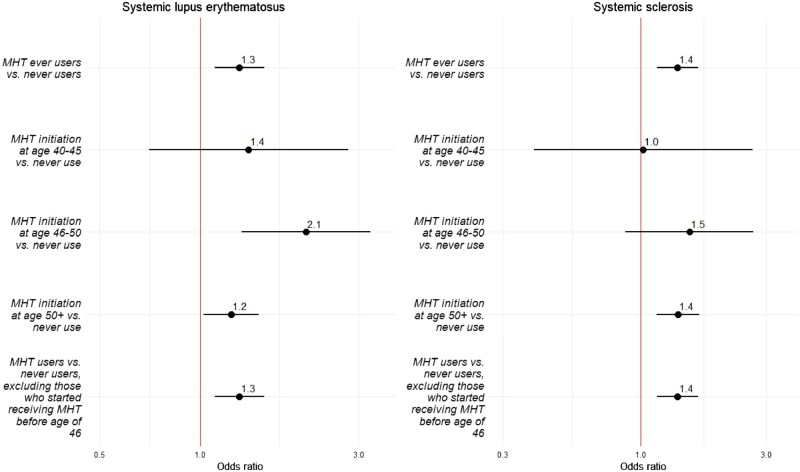
Associations between menopausal hormone therapy and SLE and SSc according to the age at first dispensation of menopausal hormone therapy. MHT, menopausal hormone therapy. Forest plot of odds ratios and their respective 95% CI distributions according to age at first dispensation of menopausal hormone therapy dispensation. The vertical dashed line denotes odds ratio of 1.0. Conditional logistic models were adjusted for years of education and gross yearly income during the calendar year before index date, sick leave (yes *vs* no) during 2005

Stratifying analysis by time from the first MHT dispensation to the index date suggested that odds ratios remained relatively stable around 2 up until 7 years before SLE diagnosis, at which point they reached an OR of 1.5 (95% CI: 1.1–1.9) (Fig. 1). Additionally, women who first received MHT at the age of 46–50 years old exhibited the highest odds of SLE (OR = 2.1; 95% CI: 1.3–3.3), followed by women who initiated MHT at the age of 40–45 years old (OR = 1.4; 95% CI: 0.7–2.8), although this association was not statistically significant (Fig. 2). Receiving MHT after 50 was associated with the lowest odds of developing MHT (OR = 1.2; 95% CI: 1.0–1.5) (Fig. 2). Exclusion of women who started receiving MHT between ages 40 and 45 years old did not attenuate association between MHT and SLE (Fig. 2).

Sensitivity analyses exploring the impact of potential misclassification of MHT showed that the associations were attenuated when restricting exposure definition to at least two dispensations with the exception of systemic oestrogen-progestogen combination treatment (Supplementary Table S3, available at *Rheumatology* online). Excluding women on tibolone and those with non-menopause indications for MHT did not fundamentally change associations with SLE (Supplementary Tables S3 and S4, available at *Rheumatology* online).

### SSc and MHT use

We included 733 women with SSc and 6571 controls (Table 2, Supplementary Fig. S2, available at *Rheumatology* online). Similarly to women with SLE, characteristics between cases and controls were similar, but women with SSc were more likely to have lower disposable income and to have taken sick leave compared with controls (Table 2). Moreover, the proportion of MHT users was higher among women with SSc compared with controls (34% *vs* 29%; Table 3). Adjusted conditional logistic regression models showed that women with a history of MHT dispensation had 1.4 (95% CI: 1.2–1.7) times the odds of SSc compared with never users (Table 3). We observed 70% increased odds of SSc associated with systemic oestrogen-progestogen combination treatments (OR = 1.7; 95% CI: 1.3–2.2), followed by local oestrogen (OR = 1.3; 95% CI: 1.0–1.6), whereas systemic oestrogen only was not associated with SSc (OR = 1.0; 95% CI: 0.6–1.7) (Table 3). Systemic only and local only MHT treatments conferred OR of 1.4 (95% CI: 1.0–1.8) and OR of 1.30 (95% CI: 1.0–1.6), respectively (Table 3). Women dispensed both local and systemic MHT medications had the highest odds of developing SSc (OR = 1.8; 95% CI: 1.2–2.5) (Table 3). In regard to duration of exposure, receiving MHT for longer than 12 months was associated with 40% increased odds of SSc (95% CI: 1.2–1.7); however, being exposed to MHT for a shorter time (<12 months) also increased the likelihood of developing SSc (OR = 1.3; 95% CI: 1.0–1.7) (Table 3).

Analyses stratified by time from the first dispensation to index date revealed that the odds associated with MHT peaked around 2–3 years before SSc diagnosis (Fig. 1). Additionally, women who started receiving MHT at ages 46–50 years old demonstrated the highest odds of SSc (OR = 1.5; 95% CI: 0.9–2.7), followed by women who started after 50 (OR = 1.4; 95% CI: 1.1–1.7) (Fig. 2). There was no association between initiating MHT treatment at 40–45 years and increased risk of SSc (OR = 1.0; 95% CI: 0.4–2.7) (Fig. 2). Moreover, the OR remained similar to main results after excluding women who started MHT treatment between ages 40 and 45 years old (Fig. 2).

Subsequent sensitivity analyses demonstrated that the association between MHT use and SSc remained robust, even after we limited exposure to at least two dispensations (Supplementary Table S3, available at *Rheumatology* online). Removing tibolone resulted in similar ORs to the main analysis (Supplementary Table S3, available at *Rheumatology* online). After women with non-menopausal indications were excluded, MHT use was still significantly associated with elevated odds of SSc (Supplementary Table S4, available at *Rheumatology* online).

## Discussion

To our knowledge, this is the first large population-based study to comprehensively examine the relationship between MHT and both SLE and SSc.

Our investigation showed an overall association between MHT use and an increased risk of SLE. Specifically, systemic oestrogen-progestogen combination treatment and dispensation of both systemic and local MHT medications increased the risk of SLE by 50–90% compared with never users. It is worth mentioning that in our study there was a limited number of women who had SLE and were on systemic oestrogen treatment, which likely contributed to overall lack of power and statistically significant associations. Notably, the risk of SLE was higher among women whose first MHT dispensation occurred at least 7 years before their SLE diagnosis and those who started receiving MHT between ages 46 and 50 years old.

In SSc, ever use of MHT was associated with 40% higher odds of developing the disease. Systemic oestrogen-progestogen combination treatment, in particular, was associated with a 70% higher risk compared with never users. However, we did not detect any statistically significant association with systemic or local oestrogen monotherapy, likely due to a small number of women who received only systemic oestrogen. Mediation through progestogen could be another reason why we did not detect any associations with systemic or local MHT treatments. Nonetheless, dispensation of both systemic and local MHT medications raised the risk of SSc by 80%. Although, it had been previously assumed that vaginal oestrogen did not produce any systemic effects, recent evidence indicates that local MHT regimens contribute to a slight elevation of circulating oestradiol [17]; this would explain the highest risk observed among women that were concurrently using both systemic and local MHT medications. Generally, women who initiated MHT ≥3 years before SSc diagnosis and those who started receiving MHT between ages 46 and 50 years old, had slightly higher odds of developing SSc than ever users of MHT (OR = 1.4 *vs* 1.5).

The effect of exogenous female sex hormones on expression of rheumatic diseases has been studied in SLE. Initial case reports suggested that MHT could lead to SLE flares among women who had been in remission [18]; however, most recent evidence points to a small increase in SLE activity associated with the use of exogenous oestrogens. In the randomized trial by Buyon *et al.* [19] the use of oral contraceptives among women with stable SLE was not associated with flares. A meta-analysis of studies that mostly excluded women with high disease activity observed significant association between exposure to MHT and elevated risk of SLE [4]. Still, the role of MHT in SLE pathogenesis remains controversial [20]. Our investigation found a significant association between MHT and increased risk of SLE. The relationship between MHT and SLE was more pronounced in the group that started MHT seven or more years before SLE diagnosis, minimizing the risk of our findings being biased by reverse causation. Initial SLE symptoms are typically non-specific and can resemble other autoimmune disorders contributing to diagnostic delay [21]. There is usually a considerable delay between the emergence of the first SLE symptoms and eventual diagnosis; the delay can be up to 2–5 years [21–23]. Therefore, disease latency could in part explain a stronger association signal in women who started MHT seven or more years before SLE diagnosis. Ovarian insufficiency could be another source of reverse causation [24–26], which was addressed by excluding women who started MHT before 40. Additional sensitivity analyses showed that excluding women with ovarian failure did not change associations between MHT and SLE. Starting MHT between 46 and 50 doubled SLE risk, while ages 40–45 did not increase risk. These findings did not support premature ovarian failure driving MHT-SLE link. However, discrepancies in risk by MHT initiation age could be also explained by earlier menopause in women with SLE [27]. Thus, the possibility of reverse causation could not be completely ruled out in our study.

Compared with SLE and RA, the relevance of exogenous female sex hormones in SSc pathogenesis has scarcely been explored. A recently published World Health Organization pharmacovigilance report underscored several novel drug classes that were suspected to increase the risk of SSc [8]. Conjugated oestrogens and medroxyprogesterone were among medications with marked disproportionality signals, accounting for 14% of all analysed individual case safety reports [8]. Our study further explored this link, demonstrating that prolonged exposure to exogenous female hormones can lead to an increased risk of SSc. We observed an even stronger association with SSc in the group that first received MHT medications at least 3 years before SSc diagnosis. Similarly to SLE, patients with SSc often experience a diagnostic delay with an average time of diagnosis ranging from 2 to 6 years [28, 29]. Paradoxically, the interval between the onset of Raynaud’s phenomenon and diagnosis of SSc was reported to be significantly longer in women compared with men [30]. Consistent with disease latency, we detected a greater magnitude of change in the risk of SSc among women who started MHT more than three years before SSc diagnosis. In contrast to SLE, premature ovarian insufficiency is relatively uncommon among patients with SSc [31] and is therefore less likely to contribute to reverse causation. We did not find sufficient evidence that premature ovarian failure was an underlying factor in the association between MHT and increased risk of SSc.

Published data suggest that some SLE-related pathogenic pathways are under the influence of oestrogen; they include changes in oestrogen and toll-like receptor signalling, cytokine secretion by T-helper and other cells, augmented autoantigen presentation and production of harmful autoantibodies [32]. Thus, MHT, which provides exogenous oestrogens, may increase the risk of developing SLE by aggravating these oestrogen-mediated pathogenic pathways.

It has been proposed that oestrogen could promote development of SSc by interacting with endothelin and inducing synthesis of several extracellular matrix proteins [33, 34]. Collectively, published data indicate that MHT increases the risk of SSc by activating key pathways underlying fibrosis [35].

The main strength of this study is its nationwide design, comprising a large number of women with SLE/SSc and matched controls from the general population. Data on MHT, medical history, socio-economic factors and additional variables used in the study, were retrieved from high-quality population-based registers, where information is entered in a prospective manner. Women with SLE/SSc were identified from routine clinical care, instead of being recruited as volunteers, which improved generalizability. In order to reduce misclassification of SLE/SSc, the definition of SLE/SSc required at least one of two visits at rheumatology/specialist unit, occurring within 1–12 month(s) from each other.

Our study had several limitations. Although the quality of the hospital register data was expected to be high, there was still a possibility of misdiagnoses. Moreover, the index date represented the first instance when women received a diagnosis, rather than the onset of the disease. Additionally, uncertainty regarding treatment adherence could lead to misclassification of the exposure. Misclassification of exposure was partially addressed in sensitivity analyses that required at least two dispensations of MHT medications, which resulted in minimal change of the OR. Additionally, because the PDR was established in 2005 [10], we have no information on MHT use before to that which might have led to the misclassification of exposure. Lifestyle-related confounders such as body mass index [36–38] and reproductive factors, including infertility and pregnancies [39, 40], were not accounted for in our analyses. Moreover, our studies were register-based and did not include any data on clinical features of SLE/SSc such as autoantibody profiles [41, 42] and clinical phenotypes [43, 44], and the generalizability of our findings was limited to women older than 40. Future investigations should explore the relationship between MHT and clinical phenotypes in SLE and SSc. Our findings indicate that MHT use might increase the risk of SLE/SSc. Future research should focus on the validation and functional interpretation of these results.

## Supplementary Material

keaf004_Supplementary_Data

## Data Availability

Summary statistics can be accessed upon request from the authors. The study data are a part of register linkage performed by Karolinska Institutet, and further sharing of these data is restricted by legal regulations.
