# Supplementary material for: Menopausal hormone therapy and the risk of systemic lupus erythematosus and systemic sclerosis: a population-based nested case-control study
Source: Rheumatology (Oxford). 2025 Jan 7;64(6):3563–70. doi: 10.1093/rheumatology/keaf004 (PMC12107026; doi:10.1093/rheumatology/keaf004)
Supplement: keaf004_Supplementary_Data [file keaf004_supplementary_data.docx]

**Supplementary material**

***Supplementary Table S1:*** *International Classification of Disease and Anatomical Therapeutic Chemical Classification codes used to identify systemic lupus erythematosus systemic sclerosis and diseases that are contraindications for menopausal hormone therapy, as well as non-menopausal indications for menopausal hormone treatment.*

| **Disease** | **International Classification of Disease codes** | **Anatomical Therapeutic Chemical Classification codes** |
| --- | --- | --- |
| ***Study Outcomes*** |  |  |
| *Systemic lupus erythematosus* | M32 and all subgroups excluding M32.0 |  |
| *Systemic sclerosis* | M34 and all subgroups excluding M34.2 |  |
| ***Menopausal hormone therapy contraindications*** |  |  |
| *Venous thromboembolism* (45) | I80, I81 and I82 | B01AA, B01AB, B01AE, B01AF, B01AX (anticoagulants) |
| *Stroke* (46) | I61, I63 and I64 |  |
| *Ischemic heart disease or acute myocardial infarction* (47) | I20–I25 |  |
|  |  |  |
| *Endometrial cancer* (48) | C54.1 |  |
| *Breast cancer* (49) | C50 |  |
| ***Non-Menopausal indications*** |  |  |
| *Premature ovarian failure* | E28.3; E89.4 |  |
| *Endometriosis* | N80.0 – N80.9 | L02AE (GnRH agonists) |
| *Premenstrual dysphoric disorder* | F32.81 |  |
| *Menstrual migraine* | G43.82; G43.83 |  |

*Data was extracted from the National Patient Register and the Prescribed Drug Register. Swedish International Classification of Disease system’s 10th revision was in use starting 1997*

***Supplementary Table S2:*** *Description of variables related to menopausal hormone therapy use and obtained from the Prescribed Drug Register.*

|  | **Explanation** |
| --- | --- |
| **Menopausal hormone therapy use** |  |
| Never | 0 dispensations of estrogen, estrogen-progestogen combination or tibolone before index date |
| Ever | 1 or more dispensations of estrogen, estrogen-progestogen combination or tibolone before index date |
| **Route of Administration** |  |
| Systemic | 1 or more dispensation of transdermal or oral MHT medications; 0 dispensations of vaginal MHT treatments before index date |
| Local | 1 or more dispensations of vaginal MHT products; 0 dispensations of transdermal or oral MHT treatments before index date |
| Systemic and local | 1 or more dispensations of vaginal products and 1 or more dispensations of oral or transdermal MHT drugs before the index date |
| Systemic estrogen | 1 or more dispensations of oral and transdermal estrogen; 0 dispensations of vaginal estrogen products, estrogen-progestogen combination and tibolone before index date |
| Local estrogen | 1 or more dispensations of vaginal estrogen; 0 dispensations of transdermal and oral estrogen products, estrogen-progestogen combination and tibolone before the index date |
| Combination of systemic estrogen and progestogen | 1 or more dispensations of oral or transdermal medications either as estrogen monotherapy or as an estrogen-progestogen combination or as tibolone before index date |
| **Duration of menopausal hormone therapy use** |  |
| Continuous variable | One dispensation was thought to continue for 4 months (3 months + 1-month carryover) and the total duration of MHT was calculated by adding up the durations of all individual dispensations. |
| <12 months |  |
| ≥12 months |  |

*First visit indicating SLE/SSc diagnosis was used as the index date*

***Supplementary Table S3:*** *Results of sensitivity analyses evaluating the impact of potential misclassification of menopausal hormone therapy on the association with systemic lupus erythematosus and systemic sclerosis.*

|  | **Menopausal hormone therapy, ≥2 dispensations vs all others at any time point prior to index date** | | | | | | **Menopausal hormone therapy, ever vs never, excluding Tibolone** | | | | | |
| --- | --- | --- | --- | --- | --- | --- | --- | --- | --- | --- | --- | --- |
|  | **Systemic lupus erythematosus** | | | **Systemic sclerosis** | | | **Systemic lupus erythematosus** | | | **Systemic sclerosis** | | |
|  | **Cases, N = 943** | **Controls, N = 8,381** | **OR (95% CI)*** | **Cases, N = 733** | **Controls, N = 6,571** | **OR (95% CI)*** | **Cases, N = 932** | **Controls, N = 8,295** | **OR (95% CI)*** | **Cases, N = 713** | **Controls, N = 6,453** | **OR (95% CI)*** |
| **Menopausal hormone therapy, n (%)** |  |  |  |  |  |  |  |  |  |  |  |  |
| *<2 dispensations* | 796 (84) | 7,228 (86) | Ref. | 578 (79) | 5,429 (83) | Ref. | 699 (75) | 6,517 (79) | Ref. | 485 (68) | 4,672 (72) | Ref. |
| *≥2 dispensations* | 147 (16) | 1,153 (14) | 1.2 (1.0 to 1.5) | 155 (21) | 1,142 (17) | 1.4 (1.1 to 1.7) | 233 (25) | 1,778 (21) | 1.3 (1.1 to 1.6) | 228 (32) | 1,781 (28) | 1.3 (1.1 to 1.6) |
| **Type of menopausal hormone therapy, n (%)^**^** |  |  |  |  |  |  |  |  |  |  |  |  |
| *systemic estrogen* | 9 (1.1) | 104 (1.4) | 0.9 (0.4 to 1.8) | 12 (2.0) | 104 (1.9) | 1.1 (0.6 to 2.1) | 19 (2.6) | 173 (2.6) | 1.0 (0.6 to 1.7) | 15 (3.0) | 161 (3.3) | 1.0 (0.6 to 1.7) |
| *local estrogen* | 83 (9.4) | 674 (8.5) | 1.2 (0.9 to 1.5) | 89 (13) | 681 (11) | 1.3 (1.0 to 1.7) | 139 (17) | 1,101 (14) | 1.3 (1.0 to 1.6) | 143 (23) | 1,152 (20) | 1.3 (1.0 to 1.6) |
| *systemic estrogen + progestogen* | 51 (6.0) | 296 (3.9) | 1.8 (1.3 to 2.4) | 45 (7.2) | 292 (5.1) | 1.6 (1.1 to 2.2) | 61 (8.0) | 401 (5.8) | 1.6 (1.2 to 2.2) | 56 (10) | 370 (7.3) | 1.6 (1.2 to 2.2) |
| **Route of administration of menopausal hormone therapy, n (%)^**^** |  |  |  |  |  |  |  |  |  |  |  |  |
| *systemic* | 28 (3.4) | 270 (3.6) | 1.1 (0.7 to 1.6) | 36 (5.9) | 265 (4.7) | 1.4 (1 to 2.0) | 50 (6.7) | 421 (6.1) | 1.2 (0.9 to 1.7) | 45 (8.5) | 385 (7.6) | 1.2 (0.9 to 1.7) |
| *local* | 83 (9.4) | 674 (8.5) | 1.2 (0.9 to 1.5) | 89 (13) | 681 (11) | 1.3 (1.0 to 1.7) | 139 (17) | 1,101 (14) | 1.3 (1.0 to 1.6) | 143 (23) | 1,152 (20) | 1.3 (1.0 to 1.6) |
| *systemic+local* | 36 (4.3) | 209 (2.8) | 1.7 (1.1 to 2.4) | 30 (4.9) | 196 (3.5) | 1.6 (1.0 to 2.4) | 44 (5.9) | 256 (3.8) | 1.8 (1.3 to 2.6) | 40 (7.6) | 244 (5.0) | 1.8 (1.3 to 2.7) |
| **Duration of MHT use, n (%)** |  |  |  |  |  |  |  |  |  |  |  |  |
| *<12 months* | 34 (3.6) | 298 (3.6) | 1.1 (0.8 to 1.6) | 41 (5.6) | 296 (4.5) | 1.4 (1.0 to 1.9) | 74 (7.9) | 546 (6.6) | 1.4 (1.0 to 1.8) | 72 (10) | 572 (8.9) | 1.3 (1.0 to 1.7) |
| *≥12 months* | 113 (12) | 855 (10) | 1.3 (1.0 to 1.6) | 114 (16) | 846 (13) | 1.4 (1.1 to 1.7) | 159 (17) | 1,232 (15) | 1.3 (1.1 to 1.6) | 156 (22) | 1,209 (19) | 1.4 (1.1 to 1.7) |

**Odds ratio (OR) and 95% confidence interval (95%CI) were estimated from conditional logistic regression models adjusted for years of education and gross yearly income during the calendar year before index date, sick leave (yes vs. no) during 2005.*

***Systemic administration was defined as oral and transdermal products (i.e. oral tablets, parenteral injections, dermal patches and dermal gel) and local as vaginal products (i.e. vaginal creams, rings and pessaries). Participants with at least 2 dispensation records of menopausal hormone therapy medications were considered as exposed, while all others were treated as unexposed.*

***Supplementary Table S4:*** *Association between menopausal hormonal therapy use and systemic lupus erythematosus and systemic sclerosis, excluding women with non-menopausal indications.*

|  | **Systemic lupus erythematosus** | | | **Systemic sclerosis** | | |
| --- | --- | --- | --- | --- | --- | --- |
|  | **Cases, N = 919** | **Controls, N = 8,216** | **OR (95% CI)*** | **Cases, N = 719** | **Controls, N = 6,474** | **OR (95% CI)*** |
| **Menopausal hormone therapy, n (%)** |  |  |  |  |  |  |
| *never* | 681 (74) | 6,394 (78) | Ref. | 473 (66) | 4,609 (71) | Ref. |
| *ever* | 238 (26) | 1,822 (22) | 1.3 (1.1 to 1.6) | 246 (34) | 1,865 (29) | 1.4 (1.2 to 1.7) |
| **Type of menopausal hormone therapy, n (%)^**^** |  |  |  |  |  |  |
| *systemic* | 47 (6.5) | 447 (6.5) | 1.1 (0.8 to 1.5) | 57 (11) | 428 (8.5) | 1.4 (1.0 to 1.9) |
| *local* | 137 (17) | 1,083 (14) | 1.3 (1.0 to 1.6) | 141 (23) | 1,138 (20) | 1.3 (1.0 to 1.6) |
| *systemic+local* | 54 (7.3) | 292 (4.4) | 1.9 (1.4 to 2.6) | 48 (9.2) | 299 (6.1) | 1.8 (1.3 to 2.5) |
| **Route of administration of menopausal hormone therapy, n (%)^**^** |  |  |  |  |  |  |
| *systemic estrogen* | 17 (2.4) | 160 (2.4) | 1.0 (0.6 to 1.7) | 15 (3.1) | 150 (3.2) | 1.1 (0.6 to 1.9) |
| *local estrogen* | 137 (17) | 1,083 (14) | 1.3 (1.0 to 1.6) | 141 (23) | 1,138 (20) | 1.3 (1.0 to 1.6) |
| *systemic estrogen + progestogen* | 71 (9.4) | 480 (7.0) | 1.6 (1.2 to 2.1) | 76 (14) | 484 (9.5) | 1.7 (1.3 to 2.3) |
| **Duration of MHT use, n (%)** |  |  |  |  |  |  |
| *<12 months* | 71 (7.7) | 539 (6.6) | 1.3 (1.0 to 1.8) | 74 (10) | 578 (8.9) | 1.3 (1.0 to 1.7) |
| *≥12 months* | 167 (18) | 1,283 (16) | 1.3 (1.1 to 1.6) | 172 (24) | 1,287 (20) | 1.4 (1.2 to 1.8) |

**Odds ratio (OR) and 95% confidence interval (95%CI) were estimated from conditional logistic regression models adjusted for years of education and gross yearly income during the calendar year before index date, sick leave (yes vs. no) during 2005.*

***Systemic administration was defined as oral and transdermal products (i.e. oral tablets, parenteral injections, dermal patches and dermal gel) and local as vaginal products (i.e. vaginal creams, rings and pessaries).*

*Participants with non-menopausal indications occurring before SLE/SSc diagnosis/index date were removed.*

**Supplementary Figures**

***Supplementary Figure S1:*** *Flowchart showing selection of individuals into the study population used to examine the risk of systemic lupus erythematosus*.


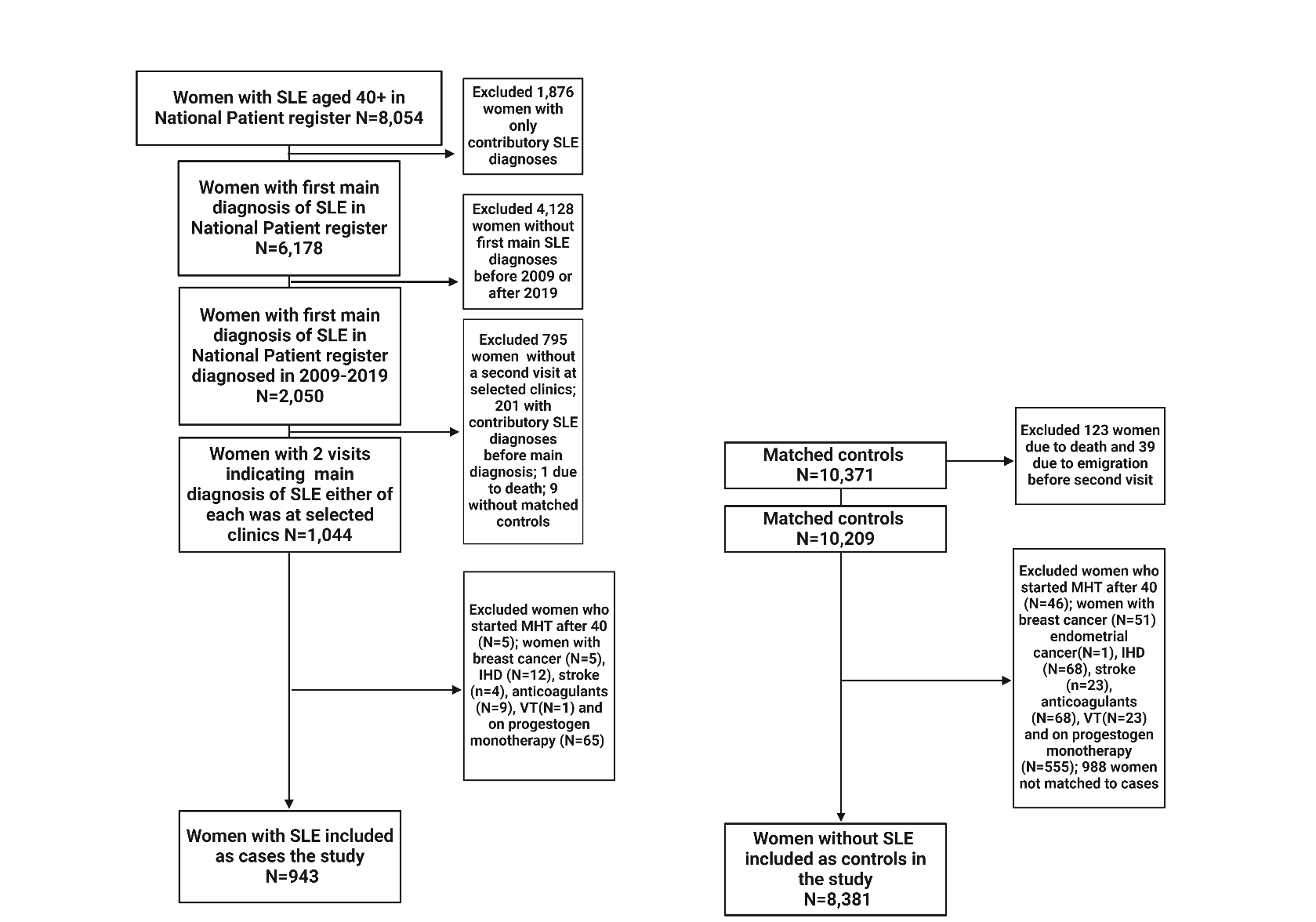


*IHD – Ischemic heart disease or acute myocardial infarction, MHT – menopausal hormone therapy, SLE – Systemic lupus erythematosus, VT – venous thromboembolism*

***Supplementary Figure S2:*** *Flowchart showing selection of individuals into the study population used to examine the risk of systemic sclerosis.*


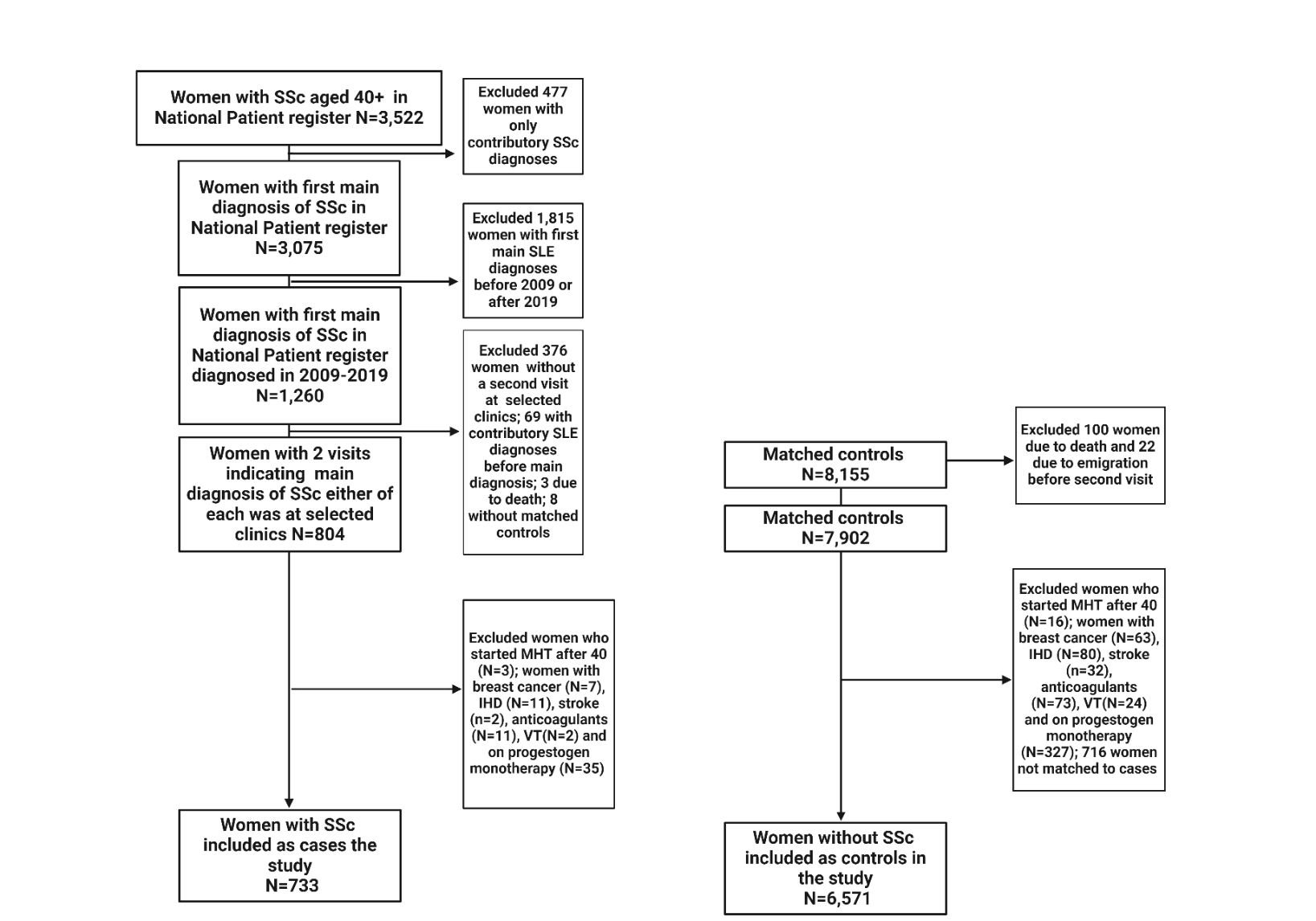


*IHD – Ischemic heart disease or acute myocardial infarction, MHT – menopausal hormone therapy, SSc – systemic sclerosis, VT – venous thromboembolis*

**Supplementary Methods**

**Supplementary Data S1**

**Assessment of menopausal hormone therapy duration**

The calculation of MHT duration incorporated number of prescriptions. Specifically, for each study participant, we calculated duration of individual prescriptions and aggregated them. We assumed that each prescription lasted 3 months, as prescriptions are required to be renewed every 3 months. We also allowed 1-month carryover, conferring 4 months (120 days) for a singular prescription. In case of overlap between two prescriptions (< 120 days between prescriptions), the duration of the first prescription was calculated from the day of its’ dispensation to the day before the dispensation of the next prescription.
